# Factors Affecting Selection of a Dialysis Modality in Elderly Patients With Chronic Kidney Disease: A Prospective Cohort Study in Korea

**DOI:** 10.3389/fmed.2022.919028

**Published:** 2022-09-27

**Authors:** Jang-Hee Cho, Jeong-Hoon Lim, Yeongwoo Park, Yena Jeon, Yon Su Kim, Shin-Wook Kang, Chul Woo Yang, Nam-Ho Kim, Hee-Yeon Jung, Ji-Young Choi, Sun-Hee Park, Chan-Duck Kim, Yong-Lim Kim

**Affiliations:** ^1^Department of Internal Medicine, School of Medicine, Kyungpook National University, Kyungpook National University Hospital, Daegu, South Korea; ^2^Clinical Research Center for End Stage Renal Disease, Daegu, South Korea; ^3^Department of Statistics, Kyungpook National University, Daegu, South Korea; ^4^Department of Internal Medicine, Seoul National University College of Medicine, Seoul, South Korea; ^5^Department of Internal Medicine, Yonsei University College of Medicine, Seoul, South Korea; ^6^Department of Internal Medicine, The Catholic University of Korea College of Medicine, Seoul, South Korea; ^7^Department of Internal Medicine, Chonnam National University Medical School, Gwangju, South Korea

**Keywords:** dialysis (ESKD), hemodialysis (HD), peritoneal dialysis (PD), aged, chronic kidney disease

## Abstract

**Background:**

We investigated factors associated with the selection of a dialysis modality for elderly patients compared to younger patients.

**Methods:**

This study included 2,514 incident dialysis patients from a Korean multicenter prospective cohort. Multivariate logistic regression analyses were performed with demographic, socioeconomic, and clinical data to analyze factors associated with the chosen dialysis modality. Differences in these factors were compared between the elderly (≥65 years) and younger (<65 years) patients.

**Results:**

Of the enrolled patients, 1,746 (69.5%) and 768 (30.6%) selected hemodialysis (HD) and peritoneal dialysis (PD), respectively. The percentage of PD was higher in younger patients than in elderly patients (37.1 vs. 16.9%, *p* < 0.001). Multivariate analysis showed that planned dialysis (*p* < 0.001), employment status (*p* < 0.001), and independent economic status (*p* = 0.048) were independent factors for selecting PD, whereas peripheral vascular disease (*p* = 0.038) and tumor (*p* = 0.010) were factors for selecting HD in the younger group. In the elderly group, planned dialysis (*p* < 0.001) and congestive heart failure (CHF; *p* = 0.002) were associated with choosing PD; however, tumor (*p* = 0.006) was associated with choosing HD. A two-way ANOVA showed that planned dialysis and CHF showed a significant interaction effect with age on modality selection.

**Conclusions:**

As the age of patients with chronic kidney disease increased, HD was more frequently selected compared to PD. Dialysis planning and CHF interacted with age in selecting dialysis modalities in elderly patients. Elderly patients were less affected by socioeconomic status than younger patients.

## Introduction

The choice of hemodialysis (HD) or peritoneal dialysis (PD) varies considerably among countries. Usually, HD is more prevalent than PD worldwide (1, 2). However, some countries have adopted PD-first policies for patients with various backgrounds and they have a much higher percentage of PD patients (3–5). This suggests that the choice of dialysis modality is associated not only with the medical condition, but also with socioeconomic factors, such as financial reimbursement or the pre-dialysis healthcare system (6, 7).

Among the many factors associated with modality selection, age exerts a strong influence on the choice of dialysis modality. Elderly patients are more prone to start with HD rather than PD compared to younger patients (1, 8). Elderly dialysis patients had a higher number of comorbidities as well as inadequate cognition and financial difficulties (9). However, most nephrologists do not consider old age as a contraindication for PD (10) and several studies failed to draw a consistent conclusion on the reasons for a decline in PD utilization with elderly patients (11–14). Furthermore, considering the diverse distributions of elderly patients receiving PD, from 6.1 to 14.2% in various countries (15, 16), non-medical factors, such as government funding (17) or the pre-dialysis delivery/education system (18), might also affect the prevalence of choosing PD. Nevertheless, there has been no study that reported factors associated with the selection of PD for elderly patients with chronic kidney disease (CKD).

With this background, we hypothesized different factors are associated with the selection of dialysis modality in elderly patients compared to younger patients. In addition, we analyzed whether the factors had any interaction with aging and aimed to identify modifiable factors related to the selection of dialysis modality.

## Materials and Methods

### Study Design and Participants

This study was performed using data from a Korean multicenter prospective cohort from August 2008 to July 2015 (The Clinical Research Center for End Stage Renal Disease [CRC for ESRD], clinicaltrial.gov NCT00931970). A total of 2,514 incident dialysis patients were enrolled from 31 dialysis centers located throughout the country. The inclusion criteria were: at least 19 years old and initiation of dialysis treatment during the study period and maintained for at least 3 months.

### Ethics Statement

All patients provided their written informed consent and the study protocol was approved by the Institutional Review Board of each center [i.e., The Catholic University of Korea, Bucheon St. Mary's Hospital; The Catholic University of Korea, Incheon St. Mary's Hospital; The Catholic University of Korea, Seoul St. Mary's Hospital; The Catholic University of Korea, St. Mary's Hospital; The Catholic University of Korea, St. Vincent's Hospital; The Catholic University of Korea, Uijeongbu St. Mary's Hospital; Cheju Halla General Hospital; Chonbuk National University Hospital; Chonnam National University Hospital; Chung-Ang University Medical Center; Chungbuk National University Hospital; Chungnam National University Hospital; Dong-A University Medical Center; Ehwa Womens University Medical Center; Fatima Hospital, Daegu; Gachon University Gil Medical Center; Inje University Pusan Paik Hospital; Kyungpook National University Hospital (2011-01-041); Kwandong University College of Medicine, Myongji Hospital; National Health Insurance Corporation Ilsan Hospital; National Medical Center; Pusan National University Hospital; Samsung Medical Center, Seoul; Seoul Metropolitan Government, Seoul National University, Boramae Medical Center; Seoul National University Hospital; Seoul National University, Bundang Hospital; Yeungnam University Medical Center; Yonsei University, Severance Hospital; Yonsei University, Gangnam Severance Hospital; Ulsan University Hospital; and Wonju Christian Hospital (in alphabetical order)]. This study was performed in accordance to the 2013 Declaration of Helsinki. Written informed consent was obtained from all patients before inclusion.

### Data Collection and Follow-Up

Demographic characteristics, referral, dialysis planning, socioeconomic situation, comorbidity diseases, blood pressure (BP), and laboratory data were recorded during study enrollment. Referral data included age at referral, sex, underlying cause of kidney disease, time from referral to dialysis, number of visits to a nephrologist, and laboratory data at referral. Planned dialysis was defined as initiation of dialysis therapy with permanent access (i.e., a PD catheter for PD or either an arteriovenous graft or a fistula for HD). The rest of dialysis initiation with a temporary or permanent catheter was considered as unplanned dialysis. Patients who underwent temporary HD due to worsening uremic symptoms during planning of PD were also classified into unplanned dialysis. Socioeconomic data included occupation, education status, medical insurance, marital status, ambulation status, degree of familial support, and smoking history. Patient ambulation status was classified into three groups as independent, partially dependent (walks with assistance and requires wheelchair), and dependent (including bed rest and immobility). Family support was the degree of support subjectively felt by the patient, and classified into three groups as none, partial support, and full support. Comorbid conditions consisted of chronic lung disease, coronary artery disease, peripheral vascular disease (PVD), cerebrovascular disease, diabetes mellitus, congestive heart failure (CHF), arrhythmia, connective tissue disease, peptic ulcer disease, sequelae of cerebrovascular disease, and tumor as well as the modified Charlson Comorbidity Index (mCCI). History of a tumor indicates the patient has previously been diagnosed any malignancy including leukemia and lymphoma (ICD-10 code: C00.x–C26.x, C30.x–C34.x, C37.x–C41.x, C43.x, C45.x–C58.x, C60.x–C85.x, C88.x, C90.x–C97.x). The laboratory data set included hemoglobin (Hb), serum albumin (Alb), and estimated glomerular filtration rate (eGFR). The eGFR was calculated using the Chronic Kidney Disease-Epidemiology Collaboration equation (19).

### Statistical Analysis

Demographic, socioeconomic, clinical, and laboratory data were compared using Pearson's chi-square test or Fisher's exact test for categorical variables and Student's *t*-test for continuous variables. Continuous variables were described as the mean ± standard deviation, and categorical variables were expressed numerically as a percentage. Patients were divided into elderly (≥65 years) and younger (<65 years) groups based on age at dialysis initiation. Factors affecting the selection of a dialysis modality were analyzed for all patients and each group. Univariate and multivariate logistic regression analyses were used to calculate the odds ratio (OR) and 95% confidence interval (CI) for selecting PD as the dialysis modality. Statistically significant variables that showed *P*-values < 0.05 in the univariate analysis were included in a multivariate analysis. The interaction of age with other factors in modality selection was tested with a two-way ANOVA. The statistical analyses were performed using SAS for Windows, version 9.2 (SAS Institute Inc., Cary, NC), and *P*-values < 0.05 were considered statistically significant.

## Results

### The Percentages of Dialysis Modalities by Age

**Figure 1:**
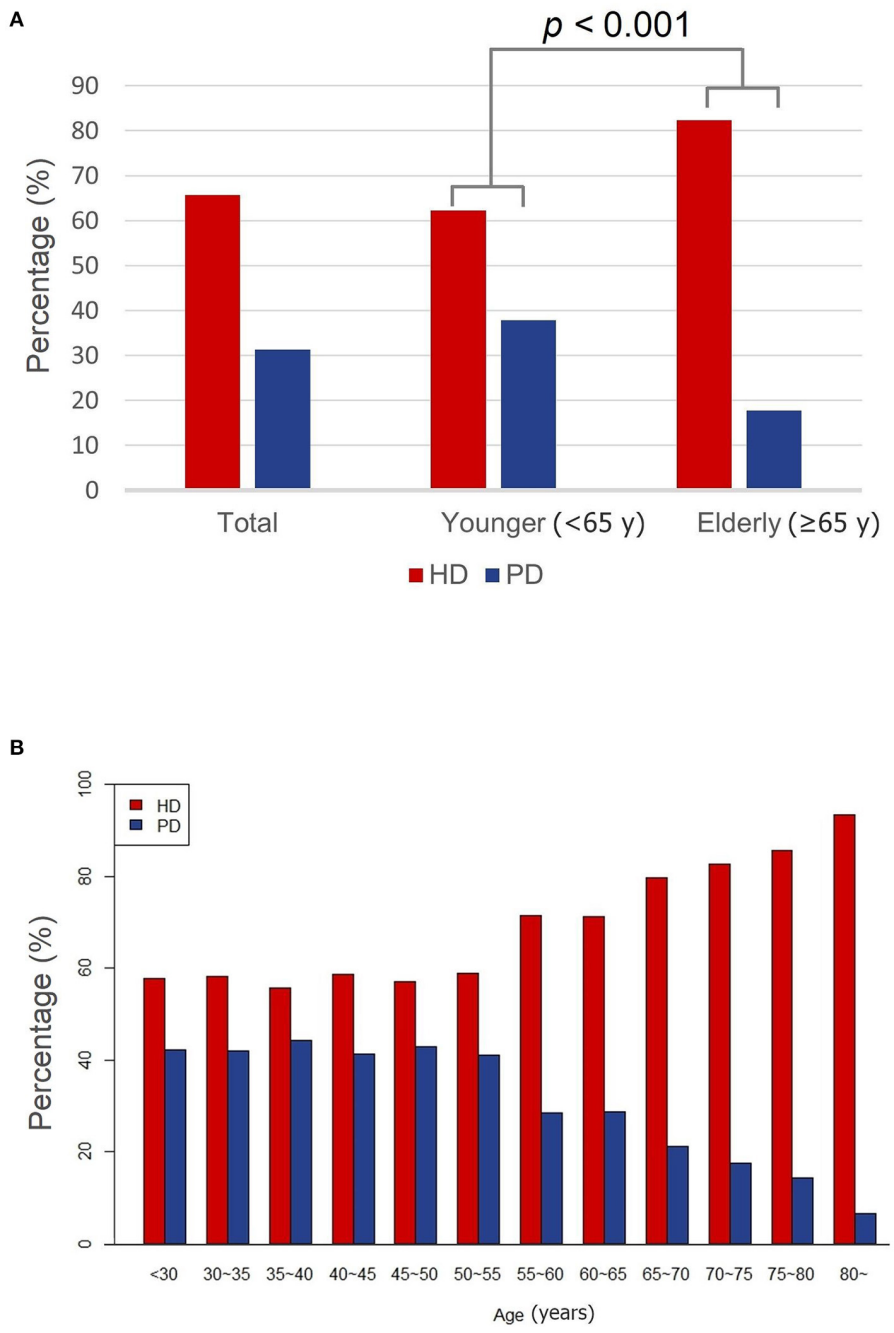
The proportion of dialysis modalities. **(A)** The proportion of dialysis modalities of younger and elderly groups. **(B)** The proportion of dialysis modalities by age group. HD, hemodialysis; PD, peritoneal dialysis; y, years.

The percentages of dialysis modalities are presented in Figure 1. Among the 2,514 enrolled patients, 1,746 (69.5%) and 768 (30.6%) selected HD and PD, respectively. The percentage of PD was higher in younger patients compared to elderly patients (37.1 vs. 16.9%, *p* < 0.001, Figure 1A). The percentage of PD was maintained at over 40% until the age of 55; thereafter it decreased gradually to 6.6% at the age over 80 (Figure 1B).

### Patient Characteristics and Factors Affecting Dialysis Modalities for all Patients

The patient characteristics for different dialysis modalities are summarized in Supplementary Table 1. At the time of referral, the mean age, underlying cause of kidney disease, mCCI, systolic BP, diastolic BP, and Hb showed a significant difference between HD and PD patients. At the initiation of dialysis, the percentage of planned dialysis, employment status, education status, ambulation status, and family support were significantly different between the two groups. PD patients had a lower prevalence of chronic lung disease, coronary artery disease, PVD, cerebrovascular disease, diabetes mellitus, arrhythmia, and history of a tumor than HD patients (all *p* < 0.05).

**Table 1:** Logistic regression analysis of factors associated with selecting peritoneal dialysis for all patients.

	**Univariate**			**Multivariate**	
	**OR (95% CI)**	***p-*value**		**aOR (95% CI)**	***p-*value**
**Findings at referral to nephrologist**					
Age	0.97 (0.96–0.97)	<0.001		1.05 (0.99–1.10)	0.056
Underlying kidney disease					
Diabetes mellitus	0.72 (0.58–0.90)	0.004		0.62 (0.34–1.14)	0.122
Glomerulonephritis	1.47 (1.10–1.97)	0.010		1.16 (0.70–1.93)	0.567
Others	Reference			Reference	
**Findings at dialysis initiation**					
Age	0.97 (0.96–0.97)	<0.001		0.93 (0.89–0.98)	0.004
modified Charlson comorbidity Index	0.82 (0.79–0.85)	<0.001		1.06 (0.94–1.19)	0.326
Hemoglobin	1.19 (1.13–1.25)	<0.001		0.94 (0.86–1.02)	0.155
Planned dialysis					
Unplanned dialysis	Reference			Reference	
Planned dialysis	40.1 (26.8–60.0)	<0.001		44.3 (26.9–72.9)	<0.001
Employment status					
Jobless including students	Reference			Reference	
Employed	2.20 (1.81–2.68)	<0.001		2.17 (1.57–2.99)	<0.001
Education					
<9 years	Reference			Reference	
10–12 years	1.25 (1.02–1.54)	0.032		0.79 (0.57–1.10)	0.167
≥13 years	1.67 (1.34–2.09)	<0.001		0.98 (0.66–1.44)	0.907
Ambulation status					
Dependent	Reference			Reference	
Partial dependent	2.90 (0.87–9.69)	0.084		0.44 (0.07–2.80)	0.385
Independent	7.09 (2.19–22.9)	0.001		0.55 (0.09–3.37)	0.517
Family support					
None	Reference			Reference	
Partial	0.70 (0.54–0.92)	0.010		0.91 (0.61–1.37)	0.652
Full support	0.45 (0.32–0.63)	<0.001		0.62 (0.37–1.04)	0.070
Comorbidity					
Chronic lung disease	0.53 (0.35–0.80)	0.003		0.83 (0.44–1.56)	0.563
Coronary artery disease	0.73 (0.56–0.95)	0.021		1.44 (0.95–2.18)	0.088
Peripheral vascular disease	0.65 (0.44–0.94)	0.023		0.76 (0.44–1.31)	0.324
Cerebrovascular disease	0.68 (0.49–0.95)	0.022		1.14 (0.67–1.96)	0.625
Diabetes	0.66 (0.56–0.78)	<0.001		1.22 (0.67–2.21)	0.516
Arrhythmia	0.51 (0.27–0.99)	0.048		0.62 (0.20–2.00)	0.417
Tumor	0.26 (0.16–0.44)	<0.001		0.28 (0.13–0.57)	<0.001

OR, odds ratio; CI, confidence interval; aOR, adjusted odds ratio.

The demographic, socioeconomic, and clinical factors for selecting PD as the dialysis modality are listed in Table 1. Multivariate analysis showed that planned dialysis (adjusted OR [aOR], 44.3; 95% CI, 26.9–72.9; *p* < 0.001) and employment status (aOR, 2.17; 95% CI, 1.57–2.99; *p* < 0.001) were associated with the selection of PD. On the contrary, old age at dialysis initiation (aOR, 0.93; 95% CI, 0.89–0.98; *p* = 0.004) and comorbidity of the history of a tumor (aOR, 0.28; 95% CI, 0.13–0.57; *p* < 0.001) were associated with the less choice of PD.

### Characteristics and Factors Affecting Dialysis Modalities of Younger Patients

The characteristics of young patients were compared between HD and PD modalities (Table 2). Age at referral, the underlying cause of kidney disease, Alb levels at referral were significantly different between the two groups. At dialysis initiation, age, mCCI, Hb, planned dialysis, employment status, education status, ambulation status, family support, comorbidities, such as chronic lung disease, PVD, diabetes mellitus, CHF, and tumor, were also significantly different between the groups.

**Table 2:** Information at referral to nephrologist and dialysis initiation by dialysis modality in younger patients.

	**Total (*n* = 1,703)**	**HD (*n* = 1,072)**	**PD (*n* = 631)**	***p-*value**
**Findings at referral to nephrologist**				
Age (years)	47.0 ± 10.8	48.0 ± 10.7	45.3 ± 10.7	<0.001
Sex, male *n* (%)	1,043 (61.2)	663 (61.9)	380 (60.2)	0.506
Underlying kidney disease, *n* (%)				<0.001
Diabetes mellitus	805 (47.5)	557 (52.3)	248 (39.4)	
Hypertension	253 (14.9)	136 (12.8)	117 (18.6)	
Glomerulonephritis	254 (15.0)	138 (13.0)	116 (18.4)	
Others	382 (22.6)	234 (22.0)	148 (23.5)	
Time from referral to dialysis (months)				0.213
>12	786 (50.4)	478 (49.1)	308 (52.4)	
≤12	775 (49.7)	495 (50.9)	280 (47.6)	
Visit number to nephrologist				0.139
<1 time	285 (17.7)	190 (18.7)	95 (15.8)	
2 times or more	1,329 (82.3)	824 (81.3)	505 (84.2)	
Hemoglobin (g/dL)	9.9 ± 2.7	9.8 ± 2.2	10.0 ± 2.4	0.059
Albumin (g/dL)	3.5 ± 0.7	3.5 ± 0.7	3.6 ± 0.7	0.024
eGFR (mL/min/1.73 m^2^)	22.1 ± 21.9	22.4 ± 23.0	21.5 ± 19.9	0.387
**Findings at dialysis initiation**				
Age (years)	49.2 ± 10.5	50.1 ± 10.5	47.7 ± 10.4	<0.001
modified Charlson Comorbidity Index	4.4 ± 2.0	4.7 ± 2.1	4.1 ± 1.9	<0.001
Hemoglobin (g/dL)	9.4 ± 1.7	9.2 ± 1.7	9.7 ± 1.7	<0.001
Albumin (g/dL)	3.5 ± 0.6	3.5 ± 0.6	3.5 ± 0.6	0.295
eGFR (mL/min/1.73 m^2^)	7.0 ± 3.5	7.0 ± 3.5	7.1 ± 3.4	0.661
Body mass index (kg/m^2^)	23.1 ± 3.5	23.2 ± 3.5	23.0 ± 3.3	0.404
Planned dialysis, *n* (%)				<0.001
Unplanned dialysis	598 (35.6)	575 (54.7)	23 (3.7)	
Planned dialysis	1,083 (64.4)	477 (45.3)	606 (96.3)	
Employment status, *n* (%)				<0.001
Jobless including students	1,092 (68.0)	736 (72.2)	356 (60.5)	
Employed	515 (32.0)	283 (27.8)	232 (39.5)	
Education				0.028
<9 years	470 (29.1)	308 (30.0)	162 (27.6)	
10–12 years	680 (42.1)	447 (43.5)	233 (39.6)	
≥13 years	466 (28.8)	273 (26.6)	193 (32.8)	
Insurance, *n* (%)				0.683
Medical aid covered for poor	441 (26.2)	274 (25.9)	167 (26.8)	
Medical insurance	1,240 (73.8)	784 (74.1)	456 (73.2)	
Marital state, *n* (%)				0.776
Single/Divorced/separated/widowed	436 (26.8)	279 (27.0)	157 (26.4)	
Married	1,191 (73.2)	753 (73.0)	438 (73.6)	
Ambulation status, *n* (%)				<0.001
Independent	1,561 (92.0)	960 (89.8)	601 (95.7)	
Partial dependent	119 (7.0)	93 (8.7)	26 (4.1)	
Dependent	17 (1.0)	16 (1.5)	1 (0.2)	
Family support, *n* (%)				0.012
None	212 (12.5)	117 (11.0)	95 (15.3)	
Partial	1,263 (74.7)	802 (75.2)	461 (74.0)	
Full support	215 (12.7)	148 (13.9)	67 (10.8)	
Smokers, *n* (%)				0.413
Never	925 (55.4)	570 (54.3)	355 (57.3)	
Current	198 (11.9)	124 (11.8)	74 (11.9)	
Former	547 (32.8)	356 (33.9)	191 (30.8)	
Comorbidity, *n* (%)				
Chronic lung disease	80 (4.7)	59 (5.5)	21 (3.3)	0.039
Coronary artery disease	147 (8.7)	101 (9.5)	46 (7.3)	0.119
Peripheral vascular disease	97 (5.7)	72 (6.8)	25 (4.0)	0.017
Cerebrovascular disease	96 (5.7)	69 (6.5)	27 (4.3)	0.060
Diabetes mellitus	898 (52.8)	611 (57.1)	287 (45.5)	<0.001
Congestive heart failure	163 (9.6)	118 (11.1)	45 (7.1)	0.008
Arrhythmia	29 (1.7)	21 (2.0)	8 (1.3)	0.284
Connective tissue disease	121 (7.1)	73 (6.8)	48 (7.6)	0.554
Peptic ulcer disease	97 (5.7)	67 (6.3)	30 (4.8)	0.192
Mild liver disease	92 (5.4)	61 (5.7)	31 (4.9)	0.484
Moderate or severe liver disease	55 (3.2)	36 (3.4)	19 (3.0)	0.692
CVA sequelae	33 (1.9)	24 (2.2)	9 (1.4)	0.230
Tumor	82 (4.8)	67 (6.3)	15 (2.4)	<0.001

HD, hemodialysis; PD, peritoneal dialysis; eGFR, estimated glomerular filtration ratio; CVA, cerebrovascular accident.

The association of various factors with selection of PD is shown in Table 3. Planned dialysis (aOR, 45.0; 95% CI, 27.1–74.6; *p* < 0.001) and employment status (aOR, 2.27; 95% CI, 1.66–3.13; *p* < 0.001) were associated with selection of PD. However, full support from family (aOR, 0.57; 95% CI, 0.33–0.99; *p* = 0.048), PVD (aOR, 0.53; 95% CI, 0.29–0.97; *p* = 0.038), and tumor (aOR, 0.38; 95% CI, 0.18–0.80; *p* = 0.010) were associated with the less selection of PD in the younger group.

**Table 3:** Logistic regression analysis of factors associated with selecting peritoneal dialysis in younger patients.

	**Univariate**			**Multivariate**	
	**OR (95% CI)**	***p-*value**		**aOR (95% CI)**	***p-*value**
**Findings at referral to nephrologist**					
Age	0.98 (0.97–0.99)	<0.001		1.03 (0.98–1.08)	0.241
Underlying kidney disease					
Diabetes mellitus	0.70 (0.55–0.91)	0.007		0.68 (0.38–1.22)	0.198
Others	Reference			Reference	
**Findings at dialysis initiation**					
Age	0.98 (0.97–0.99)	<0.001		0.95 (0.91–1.00)	0.051
modified Charlson Comorbidity Index	0.86 (0.82–0.91)	<0.001		1.03 (0.91–1.16)	0.645
Hemoglobin	1.20 (1.13–1.27)	<0.001		0.95 (0.87–1.04)	0.261
Planned dialysis					
Unplanned dialysis	Reference			Reference	
Planned dialysis	31.8 (20.6–49.0)	<0.001		45.0 (27.1–74.6)	<0.001
Employment status					
Jobless including students	Reference			Reference	
Employed	1.70 (1.37–2.10)	<0.001		2.27 (1.66–3.13)	<0.001
Education					
<9 years	Reference			Reference	
10–12 years	0.99 (0.77–1.27)	0.943		0.75 (0.53–1.08)	0.124
≥13 years	1.35 (1.04–1.76)	0.027		0.88 (0.57–1.36)	0.554
Ambulation status					
Dependent	Reference			Reference	
Independent	10.0 (1.32–75.7)	0.026		0.58 (0.05–6.22)	0.652
Family support					
None	Reference			Reference	
Partial	0.71 (0.53–0.95)	0.020		0.81 (0.53–1.24)	0.337
Full support	0.56 (0.38–0.83)	0.004		0.57 (0.33–0.99)	0.048
Comorbidity					
Peripheral vascular disease	0.57 (0.36–0.91)	0.018		0.53 (0.29–0.97)	0.038
Diabetes mellitus	0.63 (0.51–0.76)	<0.001		0.84 (0.47–1.50)	0.547
Congestive heart failure	0.62 (0.43–0.89)	0.009		0.80 (0.48–1.34)	0.405
Tumor	0.37 (0.21–0.65)	<0.001		0.38 (0.18–0.80)	0.010

OR, odds ratio; CI, confidence interval; aOR, adjusted odds ratio.

### Characteristics and Factors Affecting Dialysis Modalities of Elderly Patients

The characteristics of elderly dialysis patients were also compared between HD and PD selections (Table 4). At the time of referral, age (HD vs. PD; 69.6 ± 6.3 vs. 67.9 ± 5.2 years, *p* = 0.001) was younger and Alb levels (3.6 ± 0.6 g/dL vs. 3.8 ± 0.8, *p* = 0.030) were higher in PD patients. At the time of dialysis initiation, age (72.2 ± 5.3 vs. 70.6 ± 4.5 years, *p* < 0.001), Hb (9.4 ± 1.6 vs. 9.9 ± 1.7 g/dL, *p* < 0.001), eGFR (8.5 ± 4.4 vs. 9.5 ± 4.3 mL/min/1.73 m^2^, *p* = 0.012), planned dialysis (35.4 vs. 97.8%, *p* < 0.001), employment status (7.0 vs. 13.8%, *p* = 0.011), marriage status (78.7 vs. 89.1%, *p* = 0.007), CHF (11.0 vs. 20.6%, *p* = 0.002), and tumor (9.6 vs. 0.7%, *p* < 0.001) were significantly different between elderly HD and PD patients.

**Table 4:** Information at referral to nephrologist and dialysis initiation by dialysis modality in elderly patients.

	**Total (*n* = 811)**	**HD (*n* = 674)**	**PD (*n* = 137)**	***p-*value**
**Findings at referral to nephrologist**				
Age (years)	69.3 ± 6.1	69.6 ± 6.3	67.9 ± 5.2	0.001
Sex, male *n* (%)	509 (62.8)	414 (61.4)	95 (69.3)	0.081
Underlying kidney disease, *n* (%)				0.988
Diabetes mellitus	438 (54.3)	365 (54.5)	73 (53.7)	
Hypertension	166 (20.6)	138 (20.6)	28 (20.6)	
Glomerulonephritis	48 (5.96)	39 (5.82)	9 (6.62)	
Others	154 (19.1)	128 (19.1)	26 (19.1)	
Time from referral to dialysis (months)				
>12	435 (58.5)	357 (58.1)	78 (60.5)	0.613
≤12	309 (41.5)	258 (42.0)	51 (39.5)	
Visit number to nephrologist				
<1 time	116 (15.1)	100 (15.6)	16 (12.3)	0.335
2 times or more	654 (84.9)	540 (84.4)	114 (87.7)	
Hemoglobin (g/dL)	10.1 ± 2.0	10.1 ± 2.0	10.2 ± 1.7	0.252
Albumin (g/dL)	3.6 ± 0.7	3.6 ± 0.6	3.8 ± 0.8	0.030
eGFR (mL/min/1.73 m^2^)	22.0 ± 16.8	22.3 ± 17.4	20.6 ± 13.5	0.199
**Findings at dialysis initiation**				
Age (years)	71.9 ± 5.2	72.2 ± 5.3	70.6 ± 4.5	<0.001
modified Charlson Comorbidity Index	6.8 ± 1.9	6.9 ± 1.9	6.8 ± 1.9	0.573
Hemoglobin (g/dL)	9.5 ± 1.6	9.4 ± 1.6	9.9 ± 1.7	<0.001
Albumin (g/dL)	3.4 ± 0.5	3.4 ± 0.5	3.4 ± 0.6	0.997
eGFR (mL/min/1.73 m^2^)	8.6 ± 4.4	8.5 ± 4.4	9.5 ± 4.3	0.012
Body mass index (kg/m^2^)	22.9 ± 3.4	22.8 ± 3.5	23.2 ± 3.3	0.239
Planned dialysis, *n* (%)				<0.001
Unplanned dialysis	430 (53.9)	427 (64.6)	3 (2.2)	
Planned dialysis	368 (46.1)	234 (35.4)	134 (97.8)	
Employment status, *n* (%)				0.011
Jobless including students	714 (91.9)	608 (93.0)	106 (86.2)	
Employed	63 (8.1)	46 (7.0)	17 (13.8)	
Education				0.698
<9 years	421 (55.7)	354 (56.3)	67 (52.8)	
10–12 years	209 (27.7)	173 (27.5)	36 (28.4)	
≥13 years	126 (16.7)	102 (16.2)	24 (18.9)	
Insurance, *n* (%)				0.140
Medical aid covered for poor	154 (19.3)	134 (20.2)	20 (14.7)	
Medical insurance	646 (80.8)	530 (79.8)	116 (85.3)	
Marital state, *n* (%)				0.007
Single/Divorced/separated/widowed	151 (19.6)	137 (21.3)	14 (10.9)	
Married	619 (80.4)	505 (78.7)	114 (89.1)	
Ambulation status, *n* (%)				0.108
Independent	578 (71.5)	471 (70.1)	107 (78.1)	
Partial dependent	202 (25.0)	174 (25.9)	28 (20.4)	
Dependent	29 (3.6)	27 (4.0)	2 (1.5)	
Family support, *n* (%)				0.099
None	49 (6.1)	42 (6.3)	7 (5.1)	
Partial	561 (69.8)	455 (68.2)	106 (77.4)	
Full support	194 (24.1)	170 (25.5)	24 (17.5)	
Smokers, *n* (%)				0.114
Never	448 (56.6)	383 (58.3)	65 (48.5)	
Current	61 (7.7)	49 (7.5)	12 (9.0)	
Former	282 (35.7)	225 (34.3)	57 (42.5)	
Comorbidity, *n* (%)				
Chronic lung disease	70 (8.7)	62 (9.2)	8 (5.9)	0.215
Coronary artery disease	173 (21.4)	139 (20.7)	34 (25.4)	0.224
Peripheral vascular disease	67 (8.3)	55 (8.2)	12 (8.9)	0.783
Cerebrovascular disease	116 (14.4)	93 (13.8)	23 (16.9)	0.351
Diabetes mellitus	505 (62.4)	418 (62.1)	87 (63.5)	0.759
Congestive heart failure	102 (12.6)	74 (11.0)	28 (20.6)	0.002
Arrhythmia	30 (3.7)	27 (4.0)	3 (2.2)	0.309
Connective tissue disease	79 (9.8)	62 (9.2)	17 (12.5)	0.239
Peptic ulcer disease	52 (6.4)	47 (7.0)	5 (3.7)	0.152
Mild liver disease	29 (3.6)	24 (3.6)	5 (3.7)	0.999
Moderate or severe liver disease	23 (2.9)	21 (3.1)	2 (1.5)	0.402
CVA sequelae	34 (4.2)	26 (3.9)	8 (5.8)	0.291
Tumor	65 (8.1)	64 (9.6)	1 (0.7)	<0.001

HD, hemodialysis; PD, peritoneal dialysis; eGFR, estimated glomerular filtration ratio; CVA, cerebrovascular accident.

Multivariate analysis to validate independent effects of various factors on modality selection showed that planned dialysis (aOR, 101.0; 95% CI, 29.9–341.5; *p* < 0.001) and CHF (aOR, 3.38; 95% CI, 1.55–7.35; *p* = 0.002) were independent factors for selecting PD. Prevalence of the history of a tumor (OR, 0.06; 95% CI, 0.01–0.43; *p* = 0.006) was associated with less selecting PD in the elderly group (Table 5).

**Table 5:** Logistic regression analysis of factors associated with selecting peritoneal dialysis in elderly patients.

	**Univariate**			**Multivariate**	
	**OR (95% CI)**	***p-*value**		**aOR (95% CI)**	***p-*value**
**Findings at referral to nephrologist**					
Age	0.95 (0.92–0.99)	0.004		1.02 (0.92–1.13)	0.769
**Findings at dialysis initiation**					
Age	0.94 (0.90–0.98)	0.002		0.91 (0.80–1.03)	0.121
Hemoglobin	1.22 (1.09–1.37)	0.001		0.87 (0.72–1.04)	0.134
eGFR	1.05 (1.01–1.09)	0.014		1.09 (0.96–1.25)	0.197
Planned dialysis					
Unplanned dialysis	Reference			Reference	
Planned dialysis	81.5 (25.7–258.0)	<0.001		101.0 (29.9–341.5)	<0.001
Employment status					
Jobless including students	Reference			Reference	
Employed	2.12 (1.17–3.84)	0.013		1.77 (0.74–4.21)	0.200
Marital state					
Married	Reference			Reference	
Single/divorced/separated/widowed	0.45 (0.25–0.81)	0.008		0.80 (0.36–1.77)	0.576
Comorbidity					
Congestive heart failure	2.10 (1.30–3.39)	0.003		3.38 (1.55–7.35)	0.002
Tumor	0.07 (0.01–0.51)	0.009		0.06 (0.01–0.43)	0.006

OR, odds ratio; CI, confidence interval; aOR, adjusted odds ratio; eGFR, estimated glomerular filtration ratio.

### The Factor That Interacted With Age When Selecting a Dialysis Modality

**Figure 2:**
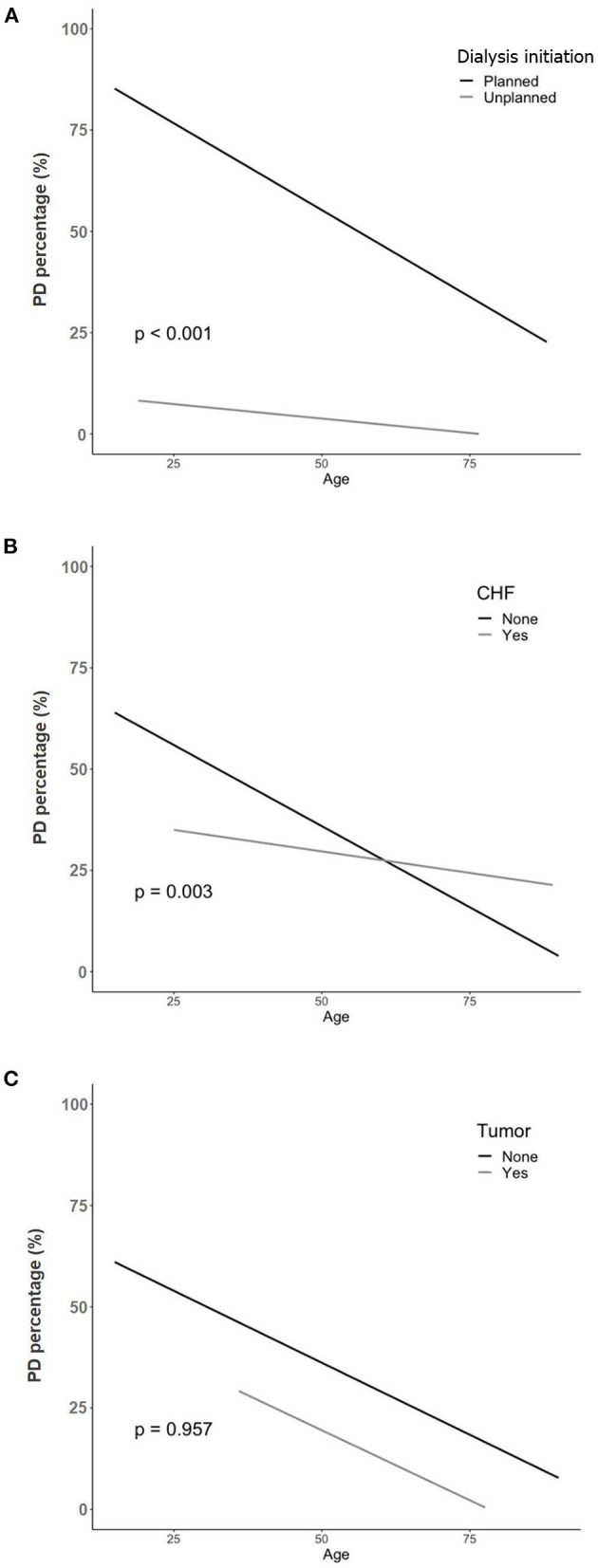
The interaction between age and other factors on selection of PD. **(A)** Age and planned dialysis. **(B)** Age and congestive heart failure. **(C)** Age and the history of a tumor. PD, peritoneal dialysis; CHF, congestive heart failure.

We identified factors that interacted with age when selecting a dialysis modality. Figure 2 displays the interaction between age and the factors. Among the factors associated with dialysis selection in elderly patients, planned dialysis and CHF showed a significant interaction effect with age and modality selection (*p* < 0.001 and *p* = 0.003, respectively).

## Discussion

This prospective multicenter cohort study investigated the factors that determined choice of dialysis modality in patients with end-stage kidney disease (ESKD) and compared the differences between younger and elderly dialysis patients. HD was more frequently selected than PD as the patient ages increased. Younger and elderly patients revealed different independent factors affecting the selection of dialysis modality. Planned dialysis, employment status, and independent economic status were independent factors related to selecting PD, whereas PVD and tumor were factors related to selecting HD in the younger group. In the elderly group, planned dialysis and CHF were associated with choosing PD; however, the history of a tumor was associated with choosing HD. Among all the factors, planned dialysis and CHF interacted with age in selecting dialysis modalities in elderly patients.

Elderly patients are more prone to start with HD rather than PD compared to younger patients, resulting in PD as the modality choice for approximately 10% of dialysis patients 65 years of age and older (1, 2, 8, 16). Similar to other reports, our study showed 16.9% of PD utilization in elderly patients and a decrease in the percentage of PD was observed over the age of 55 years. Old age was often considered a cause of non-eligibility for PD (20, 21). Several studies have evaluated eligibility for each dialysis modality and reported higher eligibility for HD than PD (8, 22–24). However, over 65% of the patients were judged medically eligible for PD in the studies and the percentage could not explain low PD penetrance in the elderly patient group (25). Rather, the combined diseases and socioeconomic situation could make the choice of PD more difficult (10, 26). This suggests that age per se is not a contraindication for PD and there is need for analysis of factors determining dialysis modality choice in elderly patients.

PD has an advantage over HD in terms of home therapy, which might be more suitable for elderly patients (27). Many of the barriers related to age could be surmounted with appropriate education (24). From this point of view, one of the important aims in our study was to determine modifiable factors related to dialysis modality choice because the selection of dialysis modality was thought to deviate to HD, especially in elderly patients.

Previous studies have explored the specific factors that contribute to choose of a specific dialysis modality. Various factors, including medical condition, referral system, patient education, resource availability, and socioeconomic status, were associated with dialysis modality selection (28–31). Our study also showed that different aspects of factors affected the selection of dialysis modality; planned dialysis and employment status were associated with the selection of PD, whereas old age and the history of a tumor were associated with the selection of HD.

The association of factors affecting the choice of dialysis modality has not been studied in an elderly patient group. As mentioned above, old age is generally known as a factor favoring HD (1, 2). Nevertheless, some elderly patients still choose PD. Thus, it is necessary to analyze factors associated with selecting PD in elderly patients and compare various factors between young and elderly patients. In our study, young patients showed an association between the selection of dialysis modality with factors such as planned dialysis, socioeconomic factors (work status and family support), and comorbid diseases (PVD and tumor). In contrast, dialysis planning and specific comorbidities, such as CHF and tumor, were associated with the selection of a dialysis modality in elderly patients. In both young and elderly patients with a history of a tumor, PD was less chosen as dialysis modality, and it might be related to the tumor-related weakness and inability to self-care, and abdominal surgical history (32). Interestingly, traditional barriers to PD, such as employment and family finances (33), were not related to modality selection in elderly patients. It is not clear how these factors relate to age, however, our data revealed that most of the elderly patients were jobless (91.9%) and received family support (93.9%), suggesting that the influence of these factors may have decreased in the group.

Furthermore, we analyzed whether the factors related to the selection of a specific modality showed an interaction with age in the elderly patient group. As age increased, the patients with planned dialysis decreased more rapidly in patients who chose PD. With an increase in age, the patients with CHF showed less decrease in the selection of PD. However, the history of a tumor did not have any interaction with age and modality selection. Planned dialysis may be difficult for elderly patients because of multiple comorbidities and obstacles (27, 34). Some studies have even reported comparable mortality rates between dialysis and conservative management in elderly CKD patients (35–37). For these reasons, for patients who cannot prepare for dialysis in a timely manner, HD might be initiated with catheter insertion and then set as maintenance therapy. Considering CHF, PD can be considered as a preferred option for elderly patients with CHF, because HD has a risk of intradialytic hypotension and hemodynamic disturbance (27, 33).

Older patients who have no contraindication to either HD or PD should be carefully evaluated for barriers to each dialysis modality. Pre-dialysis education is an integral part of care for CKD patients before reaching ESKD. Multidisciplinary pre-dialysis education could make planning for dialysis easier for elderly patients and this may improve decision-making for PD (18, 38, 39). These educational programs can further reduce the mortality of patients with CKD (40).

This study has some limitations. First, eligibility for each dialysis modality was not surveyed. The factors related to the modality selection were analyzed retrospectively after selection occurred. Second, different dialysis support policies for each country should be considered because medical policy influences the choice of dialysis modality (5). Korea does not have a PD favored policy, and different results may be obtained in regions where the ‘PD first' education program is implemented. In addition, there was a possibility of center or attending physician effect on choice of dialysis modality. Third, the type of tumor and tumor-related treatment cannot be identified by our database, and the effect of abdominal operation on selection of PD cannot be elucidated. Nevertheless, the factors associated with the selection of modality and the interaction of age with planning for dialysis and CHF in elderly dialysis patients were first analyzed and reported in the present study.

In conclusion, as the age of CKD patients increased, HD was a more frequently selected modality than PD. Dialysis planning and specific comorbidities were associated with selecting a dialysis modality in elderly patients. Elderly patients were less affected by socioeconomic status such as employment status and family support than younger patients. Clinicians should consider both medical and non-medical factors when making individualized and tailored selections about dialysis modalities.

## Data availability statement

The data analyzed in this study is subject to the following licenses/restrictions: The datasets generated during and/or analyzed during the current study are available from the corresponding author on reasonable request. Requests to access these datasets should be directed to Y-LK, ylkim@knu.ac.kr.

## Ethics statement

The studies involving human participants were reviewed and approved by Institutional Review Board of Kyungpook National University Hospital. The patients/participants provided their written informed consent to participate in this study.

## Author contributions

J-HC and Y-LK participated in research design. J-HC, YP, and YJ participated in data analysis. J-HC, J-HL, YK, S-WK, CY, N-HK, H-YJ, J-YC, S-HP, C-DK, and Y-LK participated in the performance of the research. J-HC, J-HL, and Y-LK participated in the writing of the paper and review of the paper. All authors contributed to the article and approved the submitted version.

## Funding

This research was supported by a grant of Patient-Centered Clinical Research Coordinating Center (PACEN) funded by the Ministry of Health & Welfare, Republic of Korea (HI19C0481, HC20C0054 to Y-LK), by a grant from the Korea Health Technology R&D Project through the Korea Health Industry Development Institute (KHIDI), which is funded by the Ministry of Health & Welfare, Republic of Korea (HI15C0001 to J-HC), and by a Cooperative Research Grant 2019 from the Korean Society of Nephrology (J-HC).

## Conflict of Interest

The authors declare that the research was conducted in the absence of any commercial or financial relationships that could be construed as a potential conflict of interest. The handling editor K-HO declared a shared affiliation with the author YK at the time of review.

## Publisher's Note

All claims expressed in this article are solely those of the authors and do not necessarily represent those of their affiliated organizations, or those of the publisher, the editors and the reviewers. Any product that may be evaluated in this article, or claim that may be made by its manufacturer, is not guaranteed or endorsed by the publisher.

## Supplementary material

Supplementary Table 1 Information at referral to nephrologist and dialysis initiation by dialysis modality.

## References

[1] van de Luijtgaarden MW Noordzij M Stel VS Ravani P Jarraya F Collart F. Effects of comorbid and demographic factors on dialysis modality choice and related patient survival in Europe. Nephrol Dial Transplant. (2011) 26:2940–7. 10.1093/ndt/gfq845 21325351

[2] Mehrotra R Devuyst O Davies SJ Johnson DW. The current state of peritoneal dialysis. J Am Soc Nephrol. (2016) 27:3238–52. 10.1681/ASN.2016010112 27339663 PMC5084899

[3] Ghaffari A Kalantar-Zadeh K Lee J Maddux F Moran J Nissenson A. First: peritoneal dialysis as the default transition to dialysis therapy. Semin Dial. (2013) 26:706–13. 10.1111/sdi.12125 24102745

[4] Li PK Chow KM. Peritoneal dialysis-first policy made successful: perspectives and actions. Am J Kidney Dis. (2013) 62:993–1005. 10.1053/j.ajkd.2013.03.038 23751775

[5] Liu FX Gao X Inglese G Chuengsaman P Pecoits-Filho R Yu A. Global Overview of the Impact of Peritoneal Dialysis First or Favored Policies: An Opinion. Perit Dial Int. (2015) 35:406–20. 10.3747/pdi.2013.00204 25082840 PMC4520723

[6] Li PK Chow KM Van de Luijtgaarden MW Johnson DW Jager KJ Mehrotra R. Changes in the worldwide epidemiology of peritoneal dialysis. Nat Rev Nephrol. (2017) 13:90–103. 10.1038/nrneph.2016.181 28029154

[7] Perez JJ Zhao B Qureshi S Winkelmayer WC Erickson KF. Health Insurance and the Use of Peritoneal Dialysis in the United States. Am J Kidney Dis. (2018) 71:479–87. 10.1053/j.ajkd.2017.09.024 29277511 PMC6502758

[8] Mendelssohn DC Mujais SK Soroka SD Brouillette J Takano T Barre PE. A prospective evaluation of renal replacement therapy modality eligibility. Nephrol Dial Transplant. (2009) 24:555–61. 10.1093/ndt/gfn484 18755848

[9] Brown EA Johansson L. Dialysis options for end-stage renal disease in older people. Nephron Clin Pract. (2011) 119 Suppl 1:c10–3. 10.1159/000328019 21832850

[10] Mendelssohn DC Mullaney SR Jung B Blake PG Mehta RL. What do American nephologists think about dialysis modality selection? Am J Kidney Dis. (2001) 37:22–9. 10.1053/ajkd.2001.20635 11136163

[11] Miskulin DC Meyer KB Athienites NV Martin AA Terrin N Marsh JV. Comorbidity and other factors associated with modality selection in incident dialysis patients: the CHOICE Study. Choices for Healthy Outcomes in Caring for End-Stage Renal Disease *Am J Kidney Dis*. (2002) 39:324–36. 10.1053/ajkd.2002.30552 11840373

[12] Stack AG. Determinants of modality selection among incident US dialysis patients: results from a national study. J Am Soc Nephrol. (2002) 13:1279–87. 10.1681/ASN.V1351279 11961016

[13] Xue JL Everson SE Constantini EG Ebben JP Chen SC Agodoa LY. Peritoneal and hemodialysis: II. Mortality risk associated with initial patient characteristics. Kidney Int. (2002) 61:741–6. 10.1046/j.1523-1755.2002.00176.x 11849418

[14] Mehrotra R Kermah D Fried L Kalantar-Zadeh K Khawar O Norris K. Chronic peritoneal dialysis in the United States: declining utilization despite improving outcomes. J Am Soc Nephrol. (2007) 18:2781–8. 10.1681/ASN.2006101130 17804675

[15] UK Renal Registry 23rd Annual Report: Chapter 2. Available online at: https://ukkidney.org/sites/renal.org/files/23rd_UKRR_ANNUAL_REPORT.pdf. (accessed Mar 7, 2022).

[16] U.S. Renal Data System, USRDS 2021 Annual Data Report: End Stage Renal Disease. Chapter 1. Available online at: https://adr.usrds.org/2021/end-stage-renal-disease/1-incidence-prevalence-patient-characteristics-and-treatment-modalities. (accessed Mar 7, 2022).

[17] Liu FX Treharne C Culleton B Crowe L Arici M. The financial impact of increasing home-based high dose haemodialysis and peritoneal dialysis. BMC Nephrol. (2014) 15:161. 10.1186/1471-2369-15-161 25278356 PMC4194367

[18] Schanz M Ketteler M Heck M Dippon J Alscher MD Kimmel M. Impact of an in-hospital patient education program on choice of renal replacement modality in unplanned dialysis initiation. Kidney Blood Press Res. (2017) 42:865–76. 10.1159/000484531 29161686

[19] Levey AS Stevens LA. Estimating GFR using the CKD Epidemiology Collaboration (CKD-EPI) creatinine equation: more accurate GFR estimates, lower CKD prevalence estimates, and better risk predictions. Am J Kidney Dis. (2010) 55:622–7. 10.1053/j.ajkd.2010.02.337 20338463 PMC2846308

[20] Guo A Mujais S. Patient and technique survival on peritoneal dialysis in the United States: evaluation in large incident cohorts. Kidney Int Suppl. (2003):S3–12. 10.1046/j.1523-1755.2003.08801.x 14870873

[21] Mujais S Story K. Peritoneal dialysis in the US: evaluation of outcomes in contemporary cohorts. Kidney Int Suppl. (s2006):S21–6. 10.1038/sj.ki.5001912 17080107

[22] Little J Irwin A Marshall T Rayner H Smith S. Predicting a patient's choice of dialysis modality: experience in a United Kingdom renal department. Am J Kidney Dis. (2001) 37:981–6. 10.1016/S0272-6386(05)80014-9 11325680

[23] Jager KJ Korevaar JC Dekker FW Krediet RT Boeschoten EW. The effect of contraindications and patient preference on dialysis modality selection in ESRD patients in The Netherlands. Am J Kidney Dis. (2004) 43:891–9. 10.1053/j.ajkd.2003.12.051 15112180

[24] Oliver MJ Quinn RR Richardson EP Kiss AJ Lamping DL Manns BJ. Home care assistance and the utilization of peritoneal dialysis. Kidney Int. (2007) 71:673–8. 10.1038/sj.ki.5002107 17264874

[25] Oliver MJ Quinn RR. Is the decline of peritoneal dialysis in the elderly a breakdown in the process of care? Perit Dial Int. (2008) 28:452–6. 10.1177/089686080802800505 18708535

[26] Jung B Blake PG Mehta RL Mendelssohn DC. Attitudes of Canadian nephrologists toward dialysis modality selection. Perit Dial Int. (1999) 19:263–8. 10.1177/089686089901900313 10433164

[27] Brown EA Finkelstein FO Iyasere OU Kliger AS. Peritoneal or hemodialysis for the frail elderly patient, the choice of 2 evils? Kidney Int. (2017) 91:294–303. 10.1016/j.kint.2016.08.026 27773426

[28] Smart NA Titus TT. Outcomes of early versus late nephrology referral in chronic kidney disease: a systematic review. Am J Med. 2011 124:1073-80.e2. 10.1016/j.amjmed.2011.04.026 22017785

[29] Walker RC Hanson CS Palmer SC Howard K Morton RL Marshall MR. Patient and caregiver perspectives on home hemodialysis: a systematic review. Am J Kidney Dis. (2015) 65:451–63. 10.1053/j.ajkd.2014.10.020 25582285

[30] Chiang PC Hou JJ Jong IC Hung PH Hsiao CY Ma TL. Factors Associated with the Choice of Peritoneal Dialysis in Patients with End-Stage Renal Disease. Biomed Res Int. (2016) 2016:5314719. 10.1155/2016/5314719 27042665 PMC4799809

[31] Kim HJ Park JT Han SH Yoo TH Park HC Kang SW. The pattern of choosing dialysis modality and related mortality outcomes in Korea: a national population-based study. Korean J Intern Med. (2017) 32:699–710. 10.3904/kjim.2017.141 28651309 PMC5511949

[32] Heaf J Heiro M Petersons A Vernere B Povlsen JV Sørensen AB. Choice of dialysis modality among patients initiating dialysis: results of the Peridialysis study. Clin Kidney J. (2021) 14:2064–74. 10.1093/ckj/sfaa260 34476093 PMC8406075

[33] Oliver MJ Quinn RR. Selecting peritoneal dialysis in the older dialysis population. Perit Dial Int. (2015) 35:618–21. 10.3747/pdi.2014.00346 26702000 PMC4689461

[34] Segall L Nistor I Van Biesen W Brown EA Heaf JG Lindley E. Dialysis modality choice in elderly patients with end-stage renal disease: a narrative review of the available evidence. Nephrol Dial Transplant. (2017) 32:41–9. 10.1093/ndt/gfv411 26673908

[35] Foote C Kotwal S Gallagher M Cass A Brown M Jardine M. Survival outcomes of supportive care versus dialysis therapies for elderly patients with end-stage kidney disease: A systematic review and meta-analysis. Nephrology. (2016) 21:241–53. 10.1111/nep.12586 26265214

[36] Hole B Tonkin-Crine S Caskey FJ Roderick P. Treatment of End-stage Kidney Failure without Renal Replacement Therapy. Semin Dial. (2016) 29:491–506. 10.1111/sdi.12535 27559004

[37] Engelbrecht BL Kristian MJ Inge E Elizabeth K Guldager LT Helbo TL. Does conservative kidney management offer a quantity or quality of life benefit compared to dialysis? A systematic review. BMC Nephrol. (2021) 22:307. 10.1186/s12882-021-02516-6 34507554 PMC8434727

[38] Van den Bosch J Warren DS Rutherford PA. Review of predialysis education programs: a need for standardization. Patient Prefer Adherence. (2015) 9:1279–91. 10.2147/PPA.S81284 26396500 PMC4574882

[39] Yu X Mehrotra R Yang X. Components of a successful peritoneal dialysis program. Semin Nephrol. (2017) 37:10–6. 10.1016/j.semnephrol.2016.10.003 28153189

[40] Kurella Tamura M Li S Chen SC Cavanaugh KL Whaley-Connell AT McCullough PA. Educational programs improve the preparation for dialysis and survival of patients with chronic kidney disease. Kidney Int. (2014) 85:686–92. 10.1038/ki.2013.369 24067435

