# Supplementary material for: Factors Affecting Selection of a Dialysis Modality in Elderly Patients With Chronic Kidney Disease: A Prospective Cohort Study in Korea
Source: Front Med (Lausanne). 2022 Sep 27;9:919028. doi: 10.3389/fmed.2022.919028 (PMC9550884; doi:10.3389/fmed.2022.919028)
Supplement: Supplementary Table 1 — Information at referral to nephrologist and dialysis initiation by dialysis modality. [file Table_1.PDF]

Factors affecting selection of a dialysis modality in elderly patients with chronic kidney disease: a prospective cohort study in Korea

Jang-Hee Cho[1, 2*], Jeong-Hoon Lim[1, 2*], Yeongwoo Park[2, 3], Yena Jeon[2, 3], Yon Su Kim[2, 4], Shin-Wook Kang[2, 5], Chul Woo Yang[2, 6], Nam-Ho Kim[2, 7], Hee-Yeon Jung[1, 2], Ji-Young Choi[1, 2], Sun-Hee Park[1, 2], Chan-Duck Kim[1, 2], Yong-Lim Kim[1, 2]

[1]Department of Internal Medicine, School of Medicine, Kyungpook National University, Kyungpook National University Hospital, Daegu, South Korea

[2]Clinical Research Center for End Stage Renal Disease, Daegu, South Korea

[3]Department of Statistics, Kyungpook National University, Daegu, South Korea

[4]Department of Internal Medicine, Seoul National University College of Medicine, Seoul, South Korea

[5]Department of Internal Medicine, Yonsei University College of Medicine, Seoul, South Korea

[6]Department of Internal Medicine, The Catholic University of Korea College of Medicine, Seoul, South Korea

[7]Department of Internal Medicine, Chonnam National University Medical School, Gwangju, South Korea

[*]These authors contributed equally to this work

Corresponding Author:

Yong-Lim Kim, MD, PhD

Professor

Division of Nephrology, Department of Internal Medicine

School of Medicine, Kyungpook National University, Kyungpook National University Hospital, Daegu, 41944, South Korea

Tel: +82-53-200-5553

Fax: +82-53-423-7583

E-mail: ylkim@knu.ac.kr

Supplementary Table 1. Information at referral to nephrologist and dialysis initiation by dialysis modality

	Total (n = 2,514)	HD (n = 1,746)	PD (n = 768)	*p* value
**Findings at referral to nephrologist**				
Age (years)	54.2 ± 14.1	56.4 ± 14.0	49.5 ± 13.2	<0.001
Sex, male n (%)	1552 (61.7)	1077 (61.7)	475 (61.9)	0.938
Underlying kidney disease, n (%)				<0.001
Diabetes mellitus	1243 (49.7)	922 (53.1)	321 (42.0)	
Hypertension	419 (16.8)	274 (15.8)	145 (19.0)	
Glomerulonephritis	302 (12.1)	177 (10.2)	125 (16.3)	
Others	536 (21.4)	362 (20.9)	174 (22.8)	
Time from referral to dialysis (months)				0.577
>12	1221 (53.0)	835 (52.6)	386 (53.8)	
≤12	1084 (47.0)	753 (47.4)	331 (46.2)	
Visit number to nephrologist				0.161
<1 time	401 (16.8)	290 (17.5)	111 (15.2)	
2 times or more	1983 (83.2)	1364 (82.5)	619 (84.8)	
Systolic pressure (mmHg)	144.4 ± 25.0	144.4 ± 25.2	144.5 ± 24.5	0.983
Diastolic pressure (mmHg)	82.6 ± 15.4	82.0 ± 15.4	84.23 ± 15.4	0.002
Hemoglobin (g/dL)	9.9 ± 2.2	9.9 ± 2.1	10.1 ± 2.3	0.084
Albumin (g/dL)	3.5 ± 0.7	3.5 ± 0.7	3.6 ± 0.7	0.026
eGFR (mL/min/1.73 m^2)	22.1 ± 20.4	22.4 ± 21.0	21.3 ± 18.9	0.221
**Findings at dialysis initiation**				
Age (years)	56.5 ± 14.0	58.6 ± 13.9	51.7 ± 13.1	<0.001
modified Charlson Comorbidity Index	5.2 ± 2.3	5.5 ± 2.3	4.6 ± 2.2	<0.001
Systolic pressure (mmHg)	140.9 ± 22.8	143.2 ± 22.9	135.5 ± 21.6	<0.001
Diastolic pressure (mmHg)	77.6 ± 13.9	76.9 ± 13.9	79.2 ± 14.0	<0.001
Hemoglobin (g/dL)	9.4 ± 1.7	9.3 ± 1.6	9.7 ± 1.7	<0.001
Albumin (g/dL)	3.4 ± 0.6	3.4 ± 0.6	3.5 ± 0.6	0.112
eGFR (mL/min/1.73 m^2)	7.6 ± 3.9	7.6 ± 3.9	7.5 ± 3.7	0.755
Body mass index (kg/m^2)	23.0 ± 3.5	23.0 ± 3.5	23.0 ± 3.3	0.931
Planned dialysis, n (%)				<0.001
Unplanned dialysis	1028 (41.5)	1002 (58.5)	26 (3.4)	

Planned dialysis	1451 (58.5)	711 (41.5)	740 (96.6)	
Employment status, n (%)				<0.001
Jobless including students	1806 (75.8)	1344 (80.3)	462 (65.0)	
Employed	578 (24.2)	329 (19.7)	249 (35.0)	
Education				<0.001
<9 years	891 (37.6)	662 (40.0)	229 (32.0)	
10–12 years	889 (37.5)	620 (37.4)	269 (37.6)	
≥13 years	592 (25.0)	375 (22.6)	217 (30.4)	
Insurance, n (%)				0.612
Medical aid covered for poor	595 (24.0)	408 (23.7)	187 (24.6)	
Medical insurance	1886 (76.0)	1314 (76.3)	572 (75.4)	
Marital state, n (%)				0.531
Single/Divorced/separated/widowed	587 (24.5)	416 (24.9)	171 (23.7)	
Married	1810 (75.5)	1258 (75.2)	552 (76.4)	
Ambulation status, n (%)				<0.001
Independent	2139 (85.4)	1431 (82.2)	708 (92.6)	
Partial dependent	321 (12.8)	267 (15.3)	54 (7.1)	
Dependent	46 (1.8)	43 (2.5)	3 (0.4)	
Family support, n (%)				<0.001
None	261 (10.5)	159 (9.2)	102 (13.4)	
Partial	1824 (73.1)	1257 (72.5)	567 (74.6)	
Full support	409 (16.4)	318 (18.3)	91 (12.0)	
Smokers, n (%)				0.604
Never	1373 (55.8)	953 (55.8)	420 (55.7)	
Current	259 (10.5)	173 (10.1)	86 (11.4)	
Former	829 (33.7)	581 (34.0)	248 (32.9)	
Comorbidity, n (%)				
Chronic lung disease	150 (6.0)	121 (7.0)	29 (3.8)	0.002
Coronary artery disease	320 (12.8)	240 (13.8)	80 (10.5)	0.020
Peripheral vascular disease	164 (6.6)	127 (7.3)	37 (4.8)	0.022
Cerebrovascular disease	212 (8.5)	162 (9.3)	50 (6.5)	0.021
Diabetes mellitus	1403 (55.9)	1029 (59.0)	374 (48.7)	<0.001
Congestive heart failure	265 (10.6)	192 (11.0)	73 (9.5)	0.255
Arrhythmia	59 (2.4)	48 (2.8)	11 (1.4)	0.044
Connective tissue disease	200 (8.0)	135 (7.8)	65 (8.5)	0.542
Peptic ulcer disease	149 (5.9)	114 (6.6)	35 (4.6)	0.053

Mild liver disease	121 (4.8)	85 (4.9)	36 (4.7)	0.837
Moderate or severe liver disease	78 (3.1)	57 (3.3)	21 (2.7)	0.477
CVA sequelae	67 (2.7)	50 (2.9)	17 (2.2)	0.351
Tumor	147 (5.9)	131 (7.5)	16 (2.1)	<0.001

Abbreviations: HD, hemodialysis; PD, peritoneal dialysis; eGFR, estimated glomerular filtration ratio; CVA, cerebrovascular accident.
